# Case of an Enlarged Spleen

**Published:** 1797

**Authors:** George Burrowes

**Affiliations:** M.R.I.A.


XVIII. *Caſe of an enlarged Spleen. By* George Burrowes, *M. D. M. R. I. A.*—Vide *Tranſactions of the Royal Iriſh Academy*, Vol. IV.

THE ſubject of this caſe was a man, aged about forty-four years, who, in 1792, was admitted under the author's care at the Hoſpital of the Houſe of Induſtry, in Dublin.

The patient ſaid he had laboured under dropſy for ſome months, during which time the abdomen had increaſed to the ſize it then was, which was equal, we are told, to that of a woman in the ninth month of pregnancy. His eyes were ſuffuſed with yellow, but his legs were not then, nor had they ever been, ſwelled. The abdomen was examined by ſome of the pupils at the Hoſpital, who thought they could perceive an evident fluctuation.

On the day after his admiſſion (April the 5th) he took an opening medicine, which operated well; but on the following day (the 6th) he was ſeized with ſevere vomiting and head-ach, which prevented any examination of the ſwelling, and was aſcribed to fatigue, (as he had walked ſeveral miles from the country to the Hoſpital) change of air, diet, &c.

The

The vomiting continuing on the 7th, the effervefcing draughts were prefcribed him, and fome wine. On the 8th, his pulfe was extremely quick and feeble, his fkin hot, and tongue white, and he had raved at times during the night. Blifters were then applied to the legs, an injection ordered, and the wine increafed. The vomiting ftill returned at intervals. On the 9th, all the fymptoms became much more violent, and flight hiccough difturbed him now and then. The wine was again increafed, being the only thing his ftomach would bear. On the 10th, he was in a ftate nearly approaching to coma, with conftant fingultus. Attempts were made to roufe him by farther ftimulating applications, but in vain; and he died that evening, the fixth from his admiffion, and the fourth from the attack of vomiting.

Our author examined the body the morning after his deceafe. From the circumfcribed appearance of the tumefied abdomen, he fuppofed the water might be contained in a cyft, (a circumftance, he obferves, feldom met with in men) as it much refembled ovarium dropfy, a difeafe very familiar to him in the Hofpital. On ftriking the tumour on one fide, while he kept kept his hand fixed on the other, he had no doubt of feeling a fluctuation; but when he preffed hard on the linea alba, or center of the abdomen, he was greatly furprifed, he tells us, to find a ridge reaching from the fternum to the pelvis. He then opened the cavity, and beheld the ftomach and inteftines all thrown completely to the right fide, and much diftended with air, while the whole left fide was filled up with the fpleen, enlarged to an enormous fize, and occupying the entire half of the cavity, from the diaphragm, which projected into the thorax, to the pelvis; it was neither difcoloured nor indurated, it feems, but natural in every refpect, except fize; and the veffels were not more than a third larger than in an ordinary fized fpleen.

When this enormous vifcus was taken out, it meafured, we are told, fourteen inches and a half in length, and weighed eleven pounds thirteen ounces*. The liver was fomewhat difcoloured and difeafed, but not a fpoonful of water was contained in the abdomen.

The evennefs and foft texture of the vifcus

* The ufual length is about four inches, and the weight fix or feven ounces.

on on one ſide, and the equability of the other from the diſtended inteſtines, joined to the facility with which ſuch bodies would convey the *ictus*, when ſtruck on the *left* ſide, might, the author obſerves, have deceived even an experienced ſurgeon; and had a perſon with ſuch a diſeaſe been tapped, the trocar muſt have inevitably been plunged into the viſcus, and death (from hæmorrhage) have enſued. Such a circumſtance, he adds, did once happen in Edinburgh, and the wounded ſpleen is exhibited by Dr. Monro in his lectures *.

The ſtomach was changed in poſition as well as ſite, lying more obliquely than horizontally; and ſo compreſſed was it between the liver and this enlarged ſpleen, that it required, we are told, very conſiderable force to diſtend it. That part of the colon which is tied down by a ligament, had, it ſeems, in conſequence of the great preſſure, its diameter much leſſened, and the thickneſs of its coats was very conſiderably increaſed.

It is by no means uncommon, Dr. Burrowes

* An account, by Mr. Ford, of a ſimilar accident, in a caſe where the ſpleen, after death, was found to weigh upwards of four pounds, may be ſeen in Medical Communications, Vol. II. page 137. 8vo, London, 1790. Editor.

remarks, remarks, to find this viſcus preternaturally enlarged; and inſtances of this ſort are related by various authors. Mead mentions the diſſection of a perſon in whom was found a ſpleen weighing five pounds; and Morgagni informs us of his having ſeen one of eight pounds. But that which is the ſubject of the preſent paper, ſeems to be larger than any before deſcribed.

Our ignorance of the uſes of this viſcus is conſidered as one of the diſgraces of phyſiology. Much time, our author obſerves, has been employed, and much ingenuity exhauſted, by the ancients as well as the moderns, in endeavouring to aſcertain its purpoſes in the animal economy, but without ſucceſs. Malpighi, he remarks, diſſatisfied with the conjectures of his predeceſſors, and deſpairing of ſucceeding where ſo many had failed, reſolved on an experiment from which he might rationally have expected conſiderable information. He extirpated the ſpleen in ſeveral dogs, hoping thus to diſcover by its want what had in vain been ſought for from its preſence. His ingenuity was, however, but ill requited; for though the animals lived long after the operation, and conſtant attention was paid to every circumſtance attending them, and the experiment has been been repeated in England by Mr. Boyle and others, yet it has not in any wife affifted us to difcover what are the fecretions of this gland, or what its ufe. The opinion which the ingenious Mr. Hewfon advanced a few years ago, feems to our author as wild as the conjectures of thofe who preceded him; but although the experiment of Malpighi has not difcovered to us the ufe of this vifcus, it has, however, Dr. Burrowes obferves, proved to us, that it is not *indifpenfably* neceffary to animal life; and the cafe which he has related, he thinks evinces that it may be increafed immenfely, without affecting the conftitution otherwife than by mechanical means. "Enlarged enormoufly as it "was," fays he, "it did not prevent fever, "nor did it feem to give rife to any difeafe. "Dropfy, the common confequence of ob- "ftructed or even enlarged vifcera, was not "produced by it; and had not the fever acci- "dentally come on, the man might have lived "till the preffure had prevented digeftion from "being performed." That it aggravated the fymptoms of the fever, and contributed to its danger, he will, he adds, readily confefs; but he by no means thinks that it can be confidered as a *caufe* either exciting or remote.

